# Supplementary material for: TYK2 signaling promotes the development of autoreactive CD8+ cytotoxic T lymphocytes and type 1 diabetes
Source: Nat Commun. 2024 Feb 13;15:1337. doi: 10.1038/s41467-024-45573-9 (PMC10864272; doi:10.1038/s41467-024-45573-9)
Supplement: Supplementary file 1 — Supplementary Information [file 41467_2024_45573_MOESM1_ESM.pdf]

TYK2 signaling promotes the development of autoreactive CD8$^+$ cytotoxic T lymphocytes and type 1 diabetes

Authors:

Keiichiro Mine[1,2,*], Seiho Nagafuchi[1], Satoru Akazawa[3], Norio Abiru[3,4], Hitoe Mori[1], Hironori Kurisaki[5], Kazuya Shimoda[6], Yasunobu Yoshikai[2], Hirokazu Takahashi[1,7], and Keizo Anzai[1]

Affiliations:

[1]Division of Metabolism and Endocrinology, Faculty of Medicine, Saga University, Saga, Japan.
[2]Division of Host Defense, Medical Institute of Bioregulation, Kyushu University, Fukuoka, Japan.
[3]Department of Endocrinology and Metabolism, Unit of Translational Medicine, Nagasaki University Graduate School of Biomedical Sciences, Nagasaki, Japan.
[4]Midori Clinic, Nagasaki, Japan.
[5]Department of Medical Science and Technology, Graduate School of Medical Sciences, Kyushu University, Fukuoka, Japan
[6]Division of Hematology, Diabetes, and Endocrinology, Department of Internal Medicine, Faculty of Medicine, University of Miyazaki, Miyazaki, Japan
[7]Liver Center, Saga University Hospital, Saga University, Saga, Japan.

Table of Contents:

Supplementary Figure 1……………………………………………………………………………2
Supplementary Figure 2……………………………………………………………………………4
Supplementary Figure 3……………………………………………………………………………6
Supplementary Figure 4……………………………………………………………………………8
Supplementary Figure 5……………………………………………………………………………9
Supplementary Figure 6…………………………………………………………………………...11
Supplementary Figure 7…………………………………………………………………………...13
Supplementary Table 1…………………………………………………………………………......15

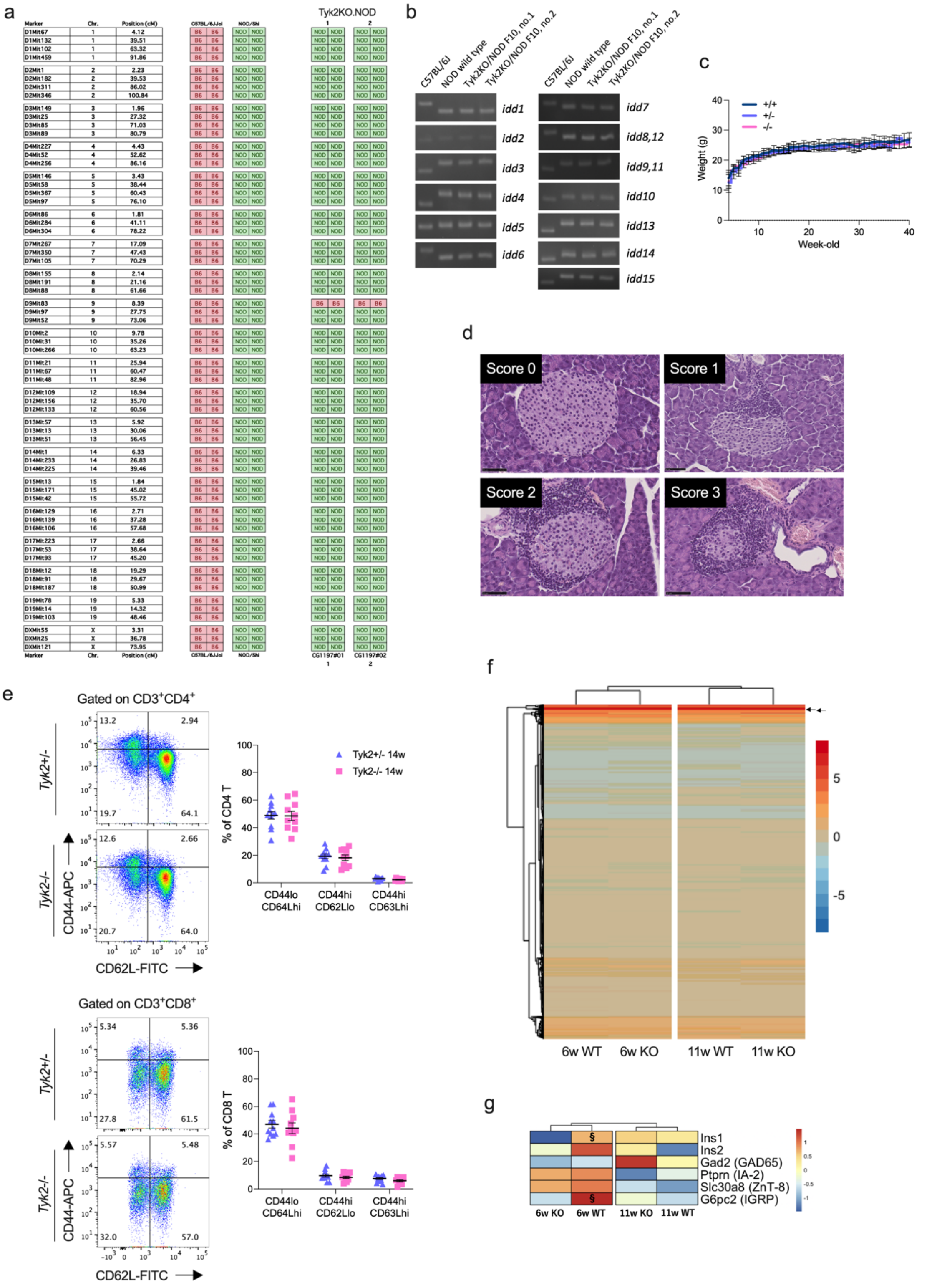

Supplementary Figure 1. Establishing *Tyk2*KO.NOD mice and the analysis of purified β-cells. **a** Analysis report of 64 short tandem repeat (STR) loci in *Tyk2*KO.NOD mice. **b** PCR analysis of STRs at the IDDM loci in *Tyk2*KO.NOD mice (Idd1 to Idd15). **c** Body weight gain in $Tyk2^{+/+}$ (n=14), $Tyk2^{+/-}$

(n=10), and *Tyk2*$^{-/-}$ NOD female mice (n=13). Diabetic mice were excluded. **d** Representative hematoxylin and eosin staining of islets with or without insulitis. Score 0, no insulitis; Score 1, peri-insulitis; Score 2, infiltrative insulitis less than 50% of the islet area; and Score 3, infiltrative insulitis more than 50% of the islet area. Bar, 50μm. **e** (Left) Representative flow cytometry plots and (right) graphs show the frequency of naïve (CD44^{lo} CD62L^{+}), effector memory (CD44^{hi} CD62L^{-}), and central memory (CD44^{hi} CD62L^{+}) in CD3^{+} CD4^{+} or CD3^{+} CD8^{+} splenocytes from *Tyk2*$^{+/-}$ (n=11) or *Tyk2*$^{-/-}$ (n=10) 14w mice. **f** Heatmap of the whole set of genes in purified β-cells. The arrows indicate the *Ins1* and *Ins2* genes, confirming the characteristics of β-cells. **g** Heatmap of the selected genes associated with islet autoantigens in purified β-cells. Criteria used to identify differentially expressed genes were as follows: upregulated genes, Z score > 2.0 and ratio < 1.5; downregulated genes, Z score < −2.0 and ratio > 0.66. *P*-values were calculated using two-tailed Student's *t*-tests (e). Data represent the mean ± SEM.

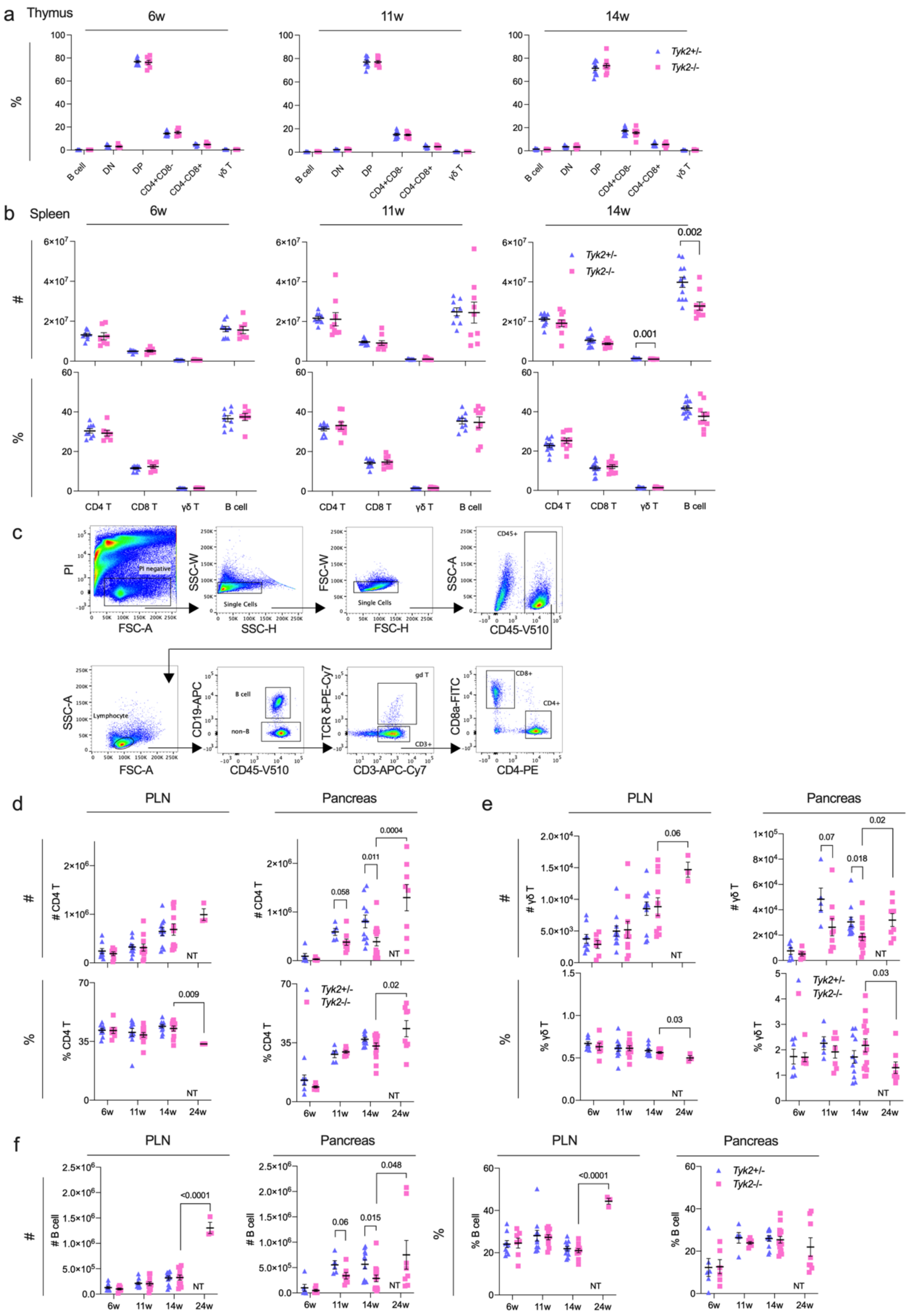

Supplementary Figure 2. Immune cell profiles in the thymus, spleen, PLN, and pancreas.

a Frequencies of the indicated immune cells in the thymi of 6w (*Tyk2*$^{+/-}$, n=9; *Tyk2*$^{-/-}$, n=7), 11w (*Tyk2*$^{+/-}$,

n=9; *Tyk2*$^{-/-}$, n=9), and 14w (*Tyk2*$^{+/-}$, n=11; *Tyk2*$^{-/-}$, n=11) female NOD mice. **b** Cell number (#) or frequency among CD45^{+} cells (%) of the indicated immune cells in the spleens of 6w (*Tyk2*$^{+/-}$, n=9; *Tyk2*$^{-/-}$, n=7), 11w (*Tyk2*$^{+/-}$, n=9; *Tyk2*$^{-/-}$, n=9), and 14w (*Tyk2*$^{+/-}$, n=12; *Tyk2*$^{-/-}$, n=10) NOD mice. **c** Gating strategy for the analysis of immune cells in the pancreas. The method described in the Materials and Methods section preserves the epitope integrity in immune cells derived from the pancreas. **d**, **e**, **and f** Cell number (#) or frequency among CD45^{+} cells (%) of CD4^{+} T cells (d), γδ T cells (e), and B cells (f) in the PLN of 6w (*Tyk2*$^{+/-}$, n=9; *Tyk2*$^{-/-}$, n=7), 11w (*Tyk2*$^{+/-}$, n=11; *Tyk2*$^{-/-}$, n=11), 14w (*Tyk2*$^{+/-}$, n=11; *Tyk2*$^{-/-}$, n=11), and normoglycemic 24w (*Tyk2*$^{-/-}$, n=3) NOD mice and pancreas of 6w (*Tyk2*$^{+/-}$, n=6; *Tyk2*$^{-/-}$, n=6), 11w (*Tyk2*$^{+/-}$, n=5; *Tyk2*$^{-/-}$, n=9), 14w (*Tyk2*$^{+/-}$, n=11; *Tyk2*$^{-/-}$, n=15), and normoglycemic 24w (*Tyk2*$^{-/-}$, n=8) NOD mice. *P*-values were calculated using two-tailed Student's *t*-tests (a, b, d-f). Data represent the mean ± SEM.

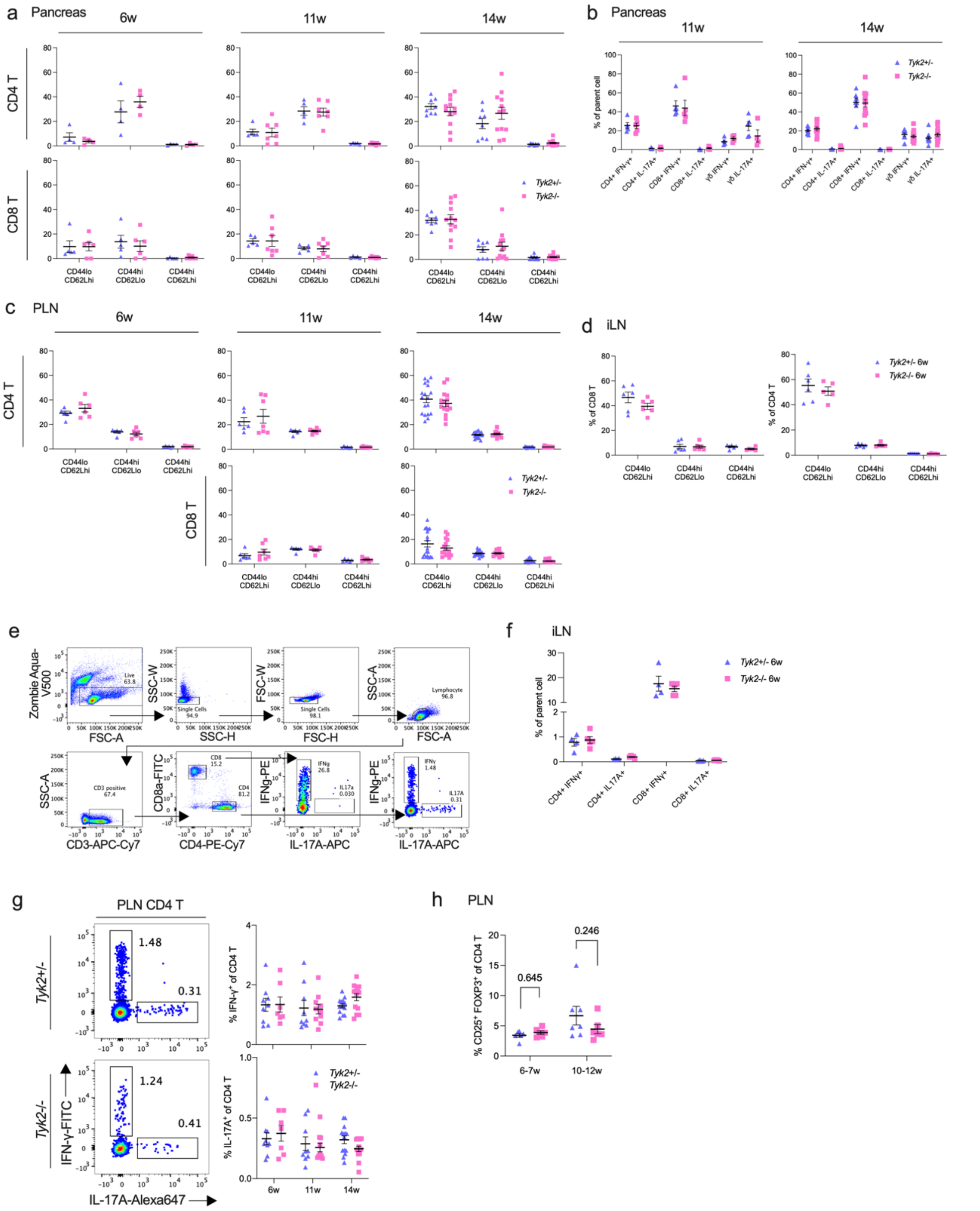

Supplementary Figure 3. Characteristics of CD4$^+$ and CD8$^+$ T cells in the PLN and pancreas.
a Frequency of naïve (CD44^{lo} CD62L$^+$), effector memory (CD44^{hi} CD62L$^-$), and central memory (CD44^{hi} CD62L$^+$) CD4$^+$ T cells in the pancreas in 6w (*Tyk2*$^{+/-}$, n=4; *Tyk2*$^{-/-}$, n=4), 11w (*Tyk2*$^{+/-}$, n=5; *Tyk2*$^{-/-}$, n=7), and 14w (*Tyk2*$^{+/-}$, n=8; *Tyk2*$^{-/-}$, n=12) NOD mice and CD8$^+$ T cells in the pancreas of 6w (*Tyk2*$^{+/-}$, n=5; *Tyk2*$^{-/-}$, n=6), 11w (*Tyk2*$^{+/-}$, n=5; *Tyk2*$^{-/-}$, n=7), and 14w (*Tyk2*$^{+/-}$, n=8; *Tyk2*$^{-/-}$, n=12) NOD mice. **b** Frequency of IFN-γ or IL-17A-producing cells upon PMA/Iono stimulation in the pancreas

of 11w (*Tyk2*$^{+/-}$, n=5; *Tyk2*$^{-/-}$, n=4 or 5) and 14w NOD mice (*Tyk2*$^{+/-}$, n=8 or 11; *Tyk2*$^{-/-}$, n=12 or 15). **c** Frequency of naïve (CD44^{lo} CD62L^{+}), effector memory (CD44^{hi} CD62L^{-}) and central memory (CD44^{hi} CD62L^{+}) CD4^{+} T cells in the PLN of 6w (*Tyk2*$^{+/-}$, n=6; *Tyk2*$^{-/-}$, n=6), 11w (*Tyk2*$^{+/-}$, n=6; *Tyk2*$^{-/-}$, n=7), and 14w (*Tyk2*$^{+/-}$, n=18; *Tyk2*$^{-/-}$, n=15) NOD mice and CD8^{+} T cells in the PLN of 11w (*Tyk2*$^{+/-}$, n=6; *Tyk2*$^{-/-}$, n=7) and 14w (*Tyk2*$^{+/-}$, n=18; *Tyk2*$^{-/-}$, n=15) NOD mice. **d** Frequency of naïve (CD44^{lo} CD62L^{+}), effector memory (CD44^{hi} CD62L^{-}), and central memory (CD44^{hi} CD62L^{+}) CD8^{+} T cells in the iLN of 6w NOD mice (*Tyk2*$^{+/-}$, n=6; *Tyk2*$^{-/-}$, n=6), and CD4^{+} T cells in the iLN of 6w NOD mice (*Tyk2*$^{+/-}$, n=6; *Tyk2*$^{-/-}$, n=5). **e** Gating strategy for the analysis of IFN-γ or IL-17A-producing cells upon PMA/Iono stimulation. **f** Frequency of IFN-γ or IL-17A-producing cells upon PMA/Iono stimulation in the iLN of 6w NOD mice (*Tyk2*$^{+/-}$, n=4; *Tyk2*$^{-/-}$, n=5). **g** (Left) Representative flow cytometry plots of 6w NOD mice and (right) frequency of IFN-γ and IL-17-producing CD4^{+} T cells upon PMA/Iono stimulation in the PLN of mice at the indicated ages. **h** Frequency of Tregs in the PLN of 6–7w (*Tyk2*$^{+/-}$, n=6; *Tyk2*$^{-/-}$, n=6) and 10–12w (*Tyk2*$^{+/-}$, n=7; *Tyk2*$^{-/-}$, n=6) NOD mice. *P*-values were calculated using two-tailed Student's *t*-tests (a-d, and f-h). Data represent the mean ± SEM.

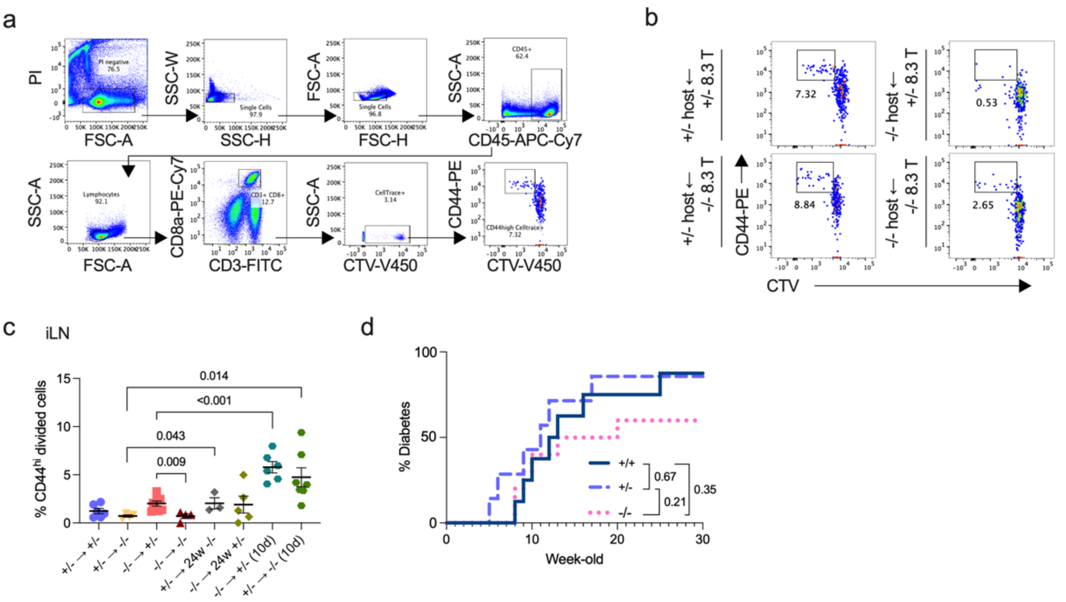

Supplementary Figure 4. The proliferation of 8.3 CD8^{+} T cells and diabetes incidence in NY8.3 NOD mice.

a Gating strategy for the analysis of CTV-positive 8.3 CD8^{+} T cells. **b** Representative flow cytometry plots of *Tyk2*$^{+/-}$ or *Tyk2*$^{-/-}$ CTV-labeled 8.3 CD8^{+} T cells in the PLN of indicated 6-9w recipient mice 5d post transfer. **c** Frequency of proliferating *Tyk2*$^{+/-}$ or *Tyk2*$^{-/-}$ CTV-labeled 8.3 CD8^{+} T cells in the iLN of the indicated recipient mice after 5d or 10d post transfer: 4×10^{6} labeled naïve 8.3 CD8^{+} T cells were transferred into 6-8w recipient mice. "24w" indicates the age of the recipient mice. (*Tyk2*$^{+/-}$ → *Tyk2*$^{+/-}$, n=6; *Tyk2*$^{-/-}$ → *Tyk2*$^{+/-}$, n=8; *Tyk2*$^{+/-}$ → *Tyk2*$^{-/-}$, n=4; *Tyk2*$^{-/-}$ → *Tyk2*$^{-/-}$, n=4; *Tyk2*$^{+/-}$ → 24w *Tyk2*$^{-/-}$, n=3; *Tyk2*$^{-/-}$ → 24w *Tyk2*$^{+/-}$, n=5; *Tyk2*$^{-/-}$ → *Tyk2*$^{+/-}$ 10d, n=6; *Tyk2*$^{+/-}$ → *Tyk2*$^{-/-}$ 10d, n=6.) **d** Incidence of spontaneous diabetes in female NY8.3 *Tyk2*$^{+/+}$ (n=8), *Tyk2*$^{+/-}$ (n=7), and *Tyk2*$^{-/-}$ NOD mice (n=10). Diabetes was defined by a non-fasting blood glucose level exceeding 250 mg/dL. *P*-values were calculated using two-tailed Student's *t*-tests (c) and log-rank test (d). Data represent the mean ± SEM.

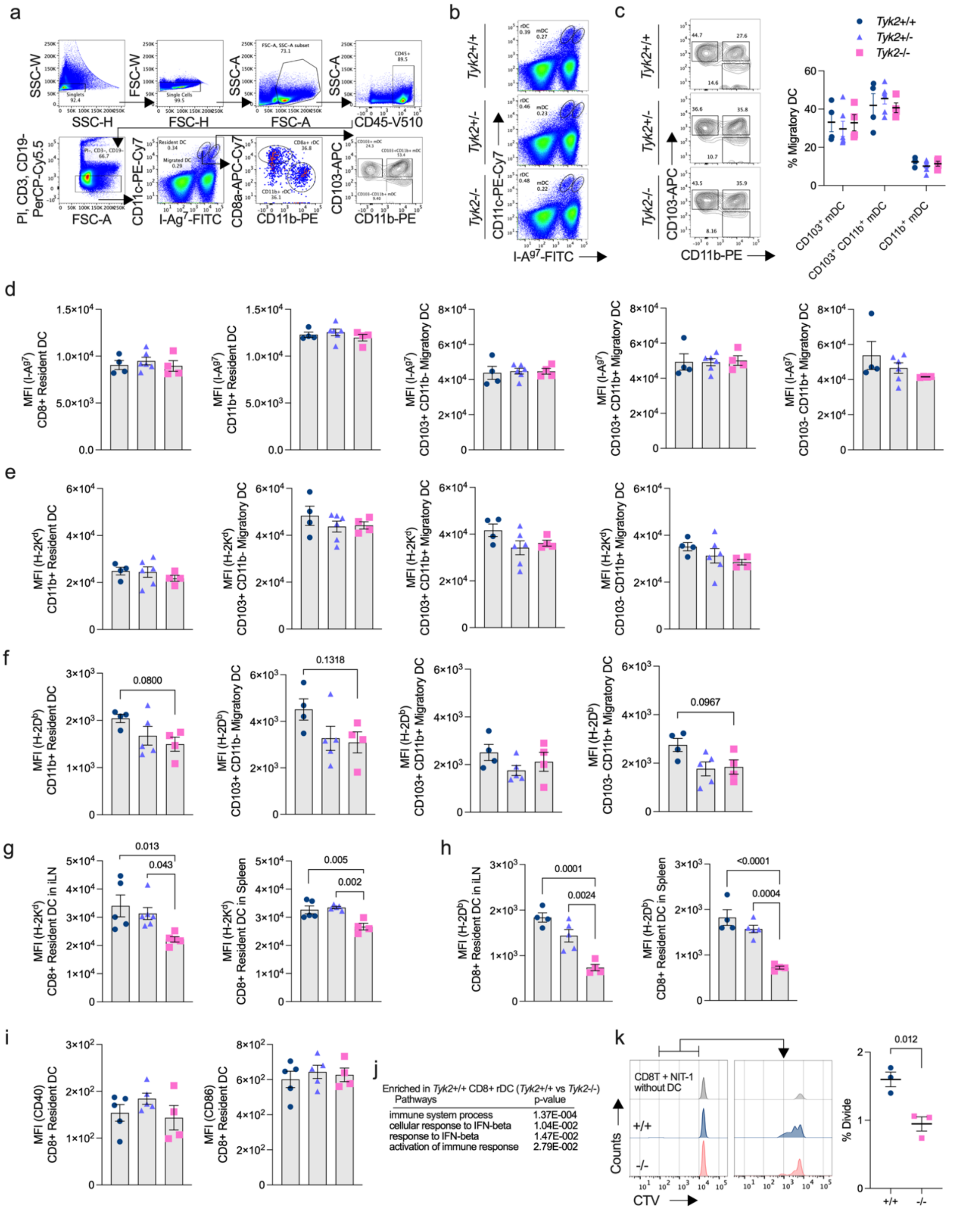

Supplementary Figure 5. Characteristics of DCs in the PLN.

a Gating strategy for the analysis and sorting of DCs. **b** Representative flow cytometry plots of DCs (resident DC, MHC II^{mid} CD11c^{hi}; migratory DC, MHC II^{hi} CD11c^{hi}) in the PLN of 6w mice. rDCs are gated on MHC II^{mid} CD11c^{hi} cells and mDCs are gated on MHC II^{hi} CD11c^{hi} cells. **c** (Left) Representatiive flow cytometry plots of mDC in the PLN of 6w mice. (Right) The frequencies of CD103^{+} CD11b^{-} mDC, CD103^{+} CD11b^{+} mDC, and CD103^{-} CD11b^{+} mDC among mDCs are shown

(*Tyk2*$^{+/+}$, n=4; *Tyk2*$^{+/-}$, n=6; *Tyk2*$^{-/-}$, n=4). **d** Expression levels of MHC II (I-A^{g7}) in the indicated types of DCs in the PLN of 6w mice (*Tyk2*$^{+/+}$, n=4; *Tyk2*$^{+/-}$, n=6; *Tyk2*$^{-/-}$, n=4). **e** Expression levels of MHC I (H-2K^{d}) in indicated types of DCs in the PLN of 6w mice (*Tyk2*$^{+/+}$, n=4; *Tyk2*$^{+/-}$, n=6; *Tyk2*$^{-/-}$, n=4). **f** Expression levels of MHC I (H-2D^{b}) in the indicated types of DCs in the PLN of 6w mice (*Tyk2*$^{+/+}$, n=4; *Tyk2*$^{+/-}$, n=6; *Tyk2*$^{-/-}$, n=4). **g** Expression levels of MHC I (H-2K^{d}) in CD8^{+} rDC in the iLN or spleen of 6w mice (*Tyk2*$^{+/+}$, n=5; *Tyk2*$^{+/-}$, n=6; *Tyk2*$^{-/-}$, n=5). **h** Expression levels of MHC I (H-2D^{b}) in CD8^{+} rDC in the iLN or spleen of 6w mice (*Tyk2*$^{+/+}$, n=5; *Tyk2*$^{+/-}$, n=6; *Tyk2*$^{-/-}$, n=5). **i** Protein expression levels of CD40 or CD86 in CD8^{+} rDCs in the PLN of 6w mice (*Tyk2*$^{+/+}$, n=5; *Tyk2*$^{+/-}$, n=5; *Tyk2*$^{-/-}$, n=4). **j** GO biological process enrichment analysis of *Tyk2*$^{+/+}$ CD8^{+} rDC vs *Tyk2*$^{-/-}$ CD8^{+} rDC in the PLN. Selected terms enriched in the *Tyk2*$^{+/+}$ CD8^{+} rDC are shown. **k** (Left) Representative histogram and (right) proliferation of CTV labeled 8.3 CD8^{+} T cells after co-culture with CD8^{+} rDC and NIT-1 cells for 3d. *P*-values were calculated using one-way ANOVA with Dunnett's posttest (c-i) and two-tailed Student's *t*-tests (k). Data represent the mean ± SEM.

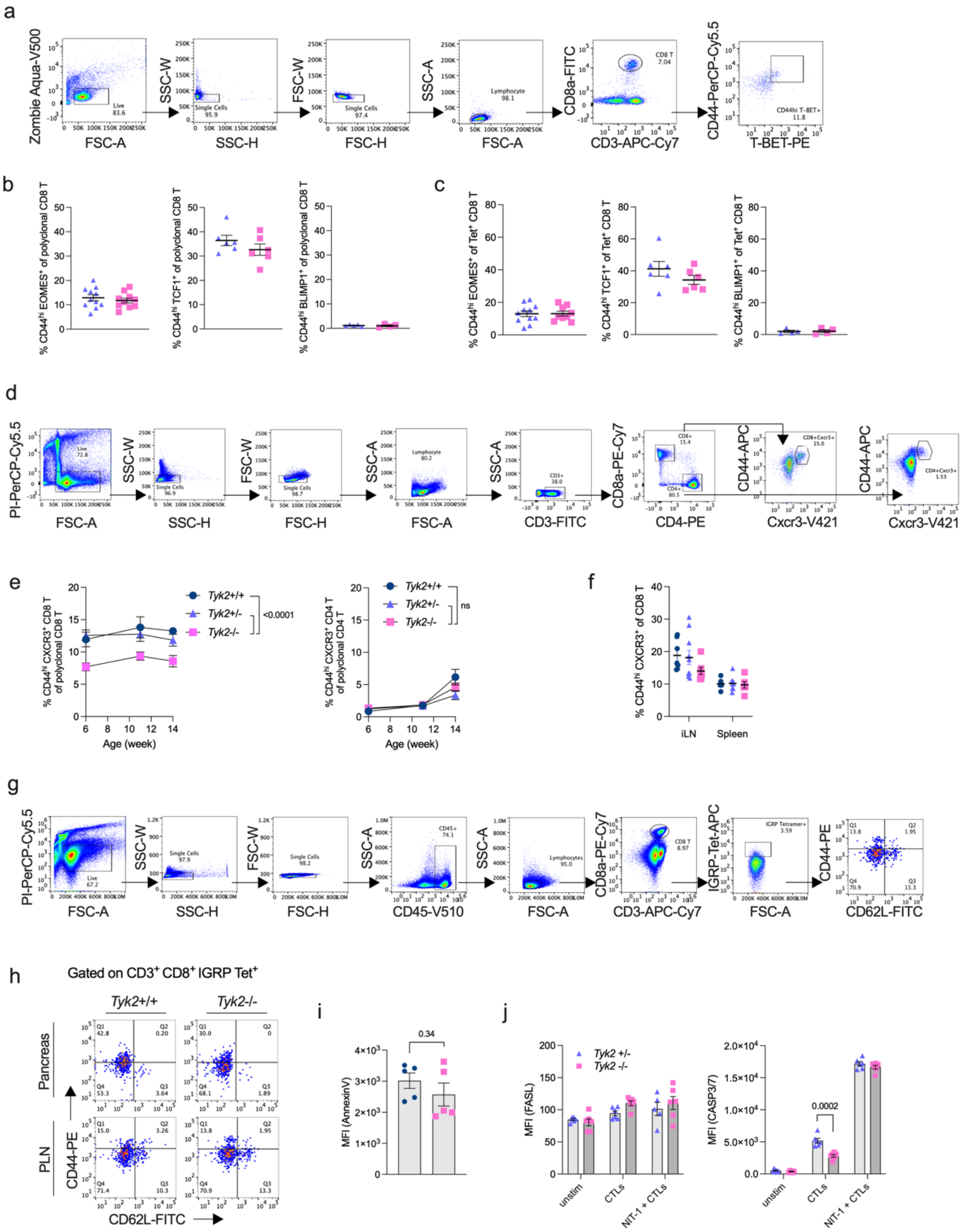

Supplementary Figure 6. Characteristics of CD8$^+$ T cells in the PLN.

a Gating strategy for the analysis of transcription factors. **b** Frequency of CD44^{hi} EOMES (*Tyk2*$^{+/-}$, n=11; *Tyk2*$^{-/-}$, n=9), TCF1 (*Tyk2*$^{+/-}$, n=6; *Tyk2*$^{-/-}$, n=6), or BLIMP1 (*Tyk2*$^{+/-}$, n=5; *Tyk2*$^{-/-}$, n=4) -positive CD8$^+$ T cells among polyclonal CD8$^+$ T cells in the PLN of 6w mice. **c** Frequency of CD44^{hi} EOMES (*Tyk2*$^{+/-}$, n=11; *Tyk2*$^{-/-}$, n=9), TCF1 (*Tyk2*$^{+/-}$, n=6; *Tyk2*$^{-/-}$, n=6), or BLIMP1 (*Tyk2*$^{+/-}$, n=5; *Tyk2*$^{-/-}$, n=4)

-positive CD8$^+$ T cells among IGRP-specific CD8$^+$ T cells in the PLN of 6w mice. **d** Gating strategy for the analysis of CXCR3 in immune cells. **e** Frequency of polyclonal CD44^{hi} CXCR3$^+$ CD8$^+$ T cells among CD8$^+$ T cells or CD44^{hi} CXCR3$^+$ CD4$^+$ T cells among CD4$^+$ T cells in the PLN (CD8$^+$ T cells from 6w (*Tyk2*$^{+/+}$, n=7; *Tyk2*$^{+/-}$, n=5; *Tyk2*$^{-/-}$, n=7); 11w (*Tyk2*$^{+/+}$, n=3; *Tyk2*$^{+/-}$, n=4; *Tyk2*$^{-/-}$, n=8); and 14w (*Tyk2*$^{+/+}$, n=3; *Tyk2*$^{+/-}$, n=7; *Tyk2*$^{-/-}$, n=4) mice; CD4$^+$ T cells from 6w (*Tyk2*$^{+/+}$, n=7; *Tyk2*$^{+/-}$, n=5; *Tyk2*$^{-/-}$, n=7); 11w (*Tyk2*$^{+/+}$, n=3; *Tyk2*$^{+/-}$, n=4; *Tyk2*$^{-/-}$, n=8); and 14w (*Tyk2*$^{+/+}$, n=3; *Tyk2*$^{+/-}$, n=4; *Tyk2*$^{-/-}$, n=7) mice). **f** Frequency of polyclonal CD44^{hi} CXCR3$^+$ CD8$^+$ T cells among CD8$^+$ T cells in the iLN (*Tyk2*$^{+/+}$, n=7; *Tyk2*$^{+/-}$, n=10; *Tyk2*$^{-/-}$, n=7) or spleen (*Tyk2*$^{+/+}$, n=5; *Tyk2*$^{+/-}$, n=8; *Tyk2*$^{-/-}$, n=6) of 6w mice. **g** Gating strategy for the analysis and sorting of IGRP-specific CD8$^+$ T cells. **h** Representative flow cytometry plots of CD44 and CD62L expressions in IGRP-specific CD8$^+$ T cells in the PLN and pancreas. **i** Annexin V binding in IGRP-specific CD8$^+$ T cells purified from the pancreas (10w *Tyk2*$^{+/+}$, n=5; 15w *Tyk2*$^{-/-}$, n=5). **j** Expression levels of FASL and active CASPASE-3/7 in unstimulated 8.3 CD8$^+$ T cells, *ex vivo*-activated 8.3 CD8$^+$ T cells, or *ex vivo*-activated 8.3 CD8$^+$ T cells cocultured with NIT-1 cells (*Tyk2*$^{+/-}$, n=6, *Tyk2*$^{-/-}$, n=6). Purified 8.3 CD8$^+$ T cells were activated with IL-2, IL-12, and CD3/28 beads for 3d. For the coculture experiment, *ex vivo*-activated 8.3 CD8$^+$ T cells were cultured with NIT-1 cells for 24h (CTLs:NIT-1=1:1). *P*-values were calculated using two-tailed Student's *t*-tests (b, c, i, and j), two-way ANOVA with Tukey's posttest (e and f). Data represent the mean ± SEM.

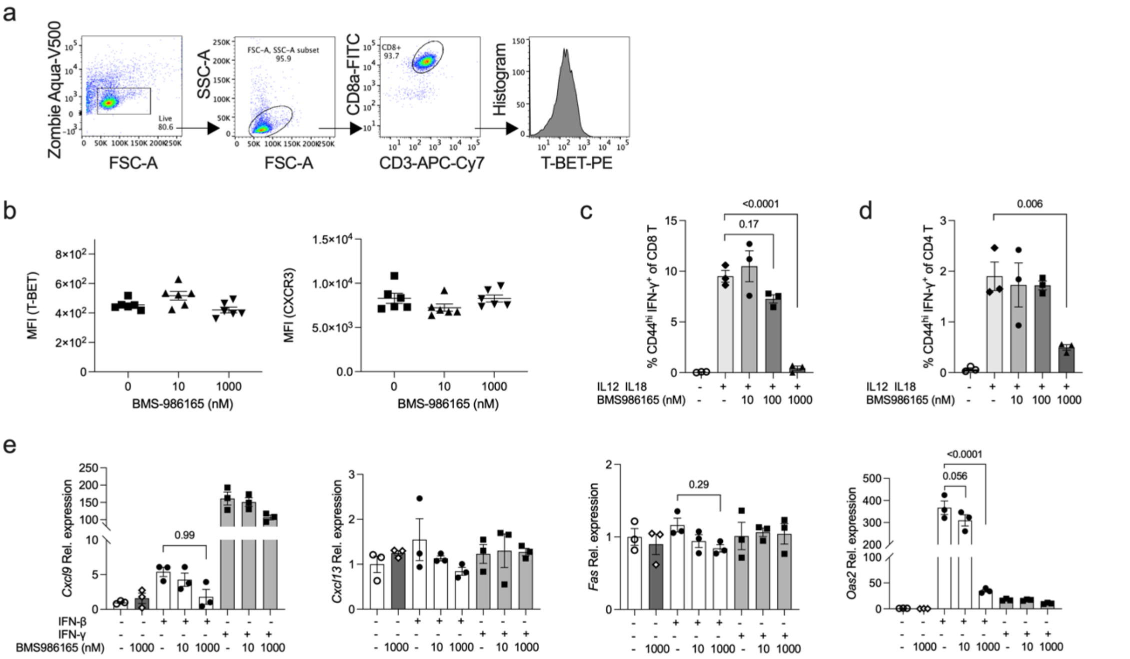

Supplementary Figure 7. Effects of BMS-986165 treatment on CTLs, $CD4^+$ T cells, and NIT-1 cells.

a Gating strategy for the analysis of T-BET expression. **b** Protein expression levels of T-BET and CXCR3 in *ex vivo*-stimulated *Tyk2*$^{+/+}$ $CD8^+$ T cells treated with BMS-986165 or vehicle for 2 days after priming. Purified $CD8^+$ T cells were activated with IL-2, IL-12, and CD3/28 beads for 3d. The activated $CD8^+$ T cells were re-cultured with BMS-986165 or vehicle for 2 days and the expressions of T-BET and CXCR3 were assessed. **c, d** Frequency of $CD44^{hi}$ IFN-γ^+ cells among *Tyk2*$^{+/+}$ $CD8^+$ T cells (c) and *Tyk2*$^{+/+}$ $CD4^+$ T cells (d) after 20 ng/mL IL-12 and 20 ng/mL IL-18 treatment in the presence or absence of BMS-986165 for 24h. **e** Gene expression levels of *Cxcl9*, *Cxcl13, Fas*, and *Oas2* in NIT-1 cells stimulated with 100 U/mL IFN-β or 0.1 ng/mL IFN-γ in the presence or absence of BMS-986165. *P*-values were calculated using one-way ANOVA with Dunnett's posttest (b-e). Data represent the mean ± SEM.

Target name	Clone name	Manufacture	Catalogue No.	Lot No.	Dilution
FITC anti-CD8a	53-6.7	BioLegend	100706	B329760	1:200
FITC anti-CD62L	MEL-14	BioLegend	104406	B324091	1:200
FITC anti-CD4	RM4-5	eBioscience	11-0042-81	2278300	1:200
FITC anti- RT1B(I-Ag7)	OX-6	BioLegend	205305	B293077	1:200
PE anti-CD4	GK1.5	BioLegend	100408	B315274	1:200
PE anti-CD3	500A2	eBioscience	12-0033-81	1911672	1:200
PE anti-FOXP3	MF-14	BioLegend	126403	B350966	1:100
PE anti-IFN-γ	XMG1.2	BioLegend	505807	B303388	1:100
PE anti-Ki67	16A8	BioLegend	652403	B293052	1:100
PE anti-T-BET	4B10	BioLegend	644809	B347808	1:100
PE anti-BLIMP-1	5E7	BioLegend	150005	B377137	1:100
PE anti-CD40	3/23	BioLegend	124609	B353789	1:200
PE anti-CD11b	M1/70	eBioscience	12-0112-82	E014719	1:200
PE anti-CD3	500A2	eBioscience	12-0033-81	1911672	1:200
PE anti-CD44	1M7	eBioscience	12-0441-81	E028485	1:200
PE anti-TCF1	C63D9	Cell Signaling Technology	C63D9	9	1:200
PE AnnexinV	-	BioLegend	640907	B405461	1:20
PerCP Cy5.5 anti-CD3	17A2	BioLegend	100217	B351063	1:200
PerCP Cy5.5 anti-GZMB	QA16A02	BioLegend	372211	B357973	1:100
PerCP Cy5.5 anti-CD44	1M7	BioLegend	103032	B213817	1:200
PerCP Cy5.5 anti-CD19	1D3	eBioscience	45-0193-80	4300340	1:200
APC anti-CD19	1D3	BioLegend	152409	B285507	1:200
APC anti-CD44	1M7	BioLegend	103012	B325365	1:200
APC anti-CD25	PC61	BioLegend	102011	B351503	1:200
APC anti-CXCR3	CXCR3-173	BioLegend	126511	B391488	1:200
APC anti-CD86	GL-1	BioLegend	105011	B346111	1:200
APC anti-CD103	2E7	BioLegend	121413	B356817	1:200
APC anti-TCR γ/δ	GL3	BioLegend	118116	B215599	1:200
APC anti-H-2Db	KH95	BioLegend	111513	B351983	1:200
APC MHC Tetramer H-2Kd	-	MBL	TB-M552-2	T2110004	1:100
Alexa Fluor 647 anti-IL17A	TC11-18H10	BD Biosciences	560184	3263593	1:100

APC-Cy7 anti-CD3	17A2	BioLegend	100222	B324939	1:200
APC-Cy7 anti-CD45	30-F11	BioLegend	103116	B345209	1:200
APC-Cy7 anti-CD8a	53-6.7	BioLegend	100713	B374507	1:200
PE-Cy7 anti-TCR γ/δ	GL3	BioLegend	118124	B291445	1:200
PE-Cy7 anti-CD8a	53-6.7	BioLegend	100721	B364216	1:200
PE-Cy7 anti-CD4	RM4-5	BioLegend	100527	B281901	1:200
PE-Cy7 anti-CD11c	N418	eBioscience	25-0114-82	2295639	1:200
PE-Cy7 anti-EOMES	Dan11mag	eBioscience	25-4875-80	2511279	1:100
BV421 anti-H2-Kd	SF-1.1	BioLegend	116623	B294310	1:200
BV421 anti-CXCR3	CXCR3-173	BioLegend	126521	B315428	1:200
BV421 anti-CD103	2E7	BioLegend	121421	B366374	1:200
BV450 anti-CD4	RM4-5	BD Biosciences	560468	5351553	1:200
BV450 anti-CD11b	M1/70	BD Biosciences	560456	50193	1:200
BV510 anti-CD45	30-F11	BioLegend	103134	B314484	1:200
Purified anti-CD16/32	93	BioLegend	101302	B320249	1:500

Supplementary Table 1. Antibodies information.
