# Supplementary material for: TYK2 signaling promotes the development of autoreactive CD8+ cytotoxic T lymphocytes and type 1 diabetes
Source: Nat Commun. 2024 Feb 13;15:1337. doi: 10.1038/s41467-024-45573-9 (PMC10864272; doi:10.1038/s41467-024-45573-9)
Supplement: Supplementary file 3 — Reporting Summary [file 41467_2024_45573_MOESM3_ESM.pdf]

nature portfolio

Corresponding author(s): Keiichiro Mine

Last updated by author(s): January 17, 2024

Reporting Summary

Nature Portfolio wishes to improve the reproducibility of the work that we publish. This form provides structure for consistency and transparency in reporting. For further information on Nature Portfolio policies, see our Editorial Policies and the Editorial Policy Checklist.

Statistics

For all statistical analyses, confirm that the following items are present in the figure legend, table legend, main text, or Methods section.

n/a	Confirmed	
☐	☒	The exact sample size (*n*) for each experimental group/condition, given as a discrete number and unit of measurement
☐	☒	A statement on whether measurements were taken from distinct samples or whether the same sample was measured repeatedly
☐	☒	The statistical test(s) used AND whether they are one- or two-sided<br>*Only common tests should be described solely by name; describe more complex techniques in the Methods section.*
☒	☐	A description of all covariates tested
☒	☐	A description of any assumptions or corrections, such as tests of normality and adjustment for multiple comparisons
☐	☒	A full description of the statistical parameters including central tendency (e.g. means) or other basic estimates (e.g. regression coefficient) AND variation (e.g. standard deviation) or associated estimates of uncertainty (e.g. confidence intervals)
☐	☒	For null hypothesis testing, the test statistic (e.g. *F*, *t*, *r*) with confidence intervals, effect sizes, degrees of freedom and *P* value noted<br>*Give P values as exact values whenever suitable.*
☒	☐	For Bayesian analysis, information on the choice of priors and Markov chain Monte Carlo settings
☒	☐	For hierarchical and complex designs, identification of the appropriate level for tests and full reporting of outcomes
☒	☐	Estimates of effect sizes (e.g. Cohen's *d*, Pearson's *r*), indicating how they were calculated

Our web collection on statistics for biologists contains articles on many of the points above.

Software and code

Policy information about availability of computer code

Data collection

Flow Cytometry: FACSVerse (BD Biosciences), MA900 (Sony)
RNA-sequencing: Illumina NovaSeq 6000 on a 150bp paired-end with a coverage of 40M reads per sample
Microarray: Affymetrix Mouse Transcriptome Array 1.0

Data analysis

Flow Cytometry: FlowJo (10.6.1)

Transcriptomics (RNA-sequencing):
-R (3.6)
-RStudio (2023.06.1+524)
-NovaSeq Control Software (1.7.5)
-Real Time Analysis (RTA) (3.4.4)
-bcl2fastq2 (2.20)
-DRAGEN Bio-IT Platform (3.9.3)
-GeneTrail (3.2)
-RNASeqChef (1.0.7)
-ggVolcanoR (https://ggvolcanor.erc.monash.edu/)

Transcriptomics (Microarray):
-R (3.6)
-RStudio (2023.06.1+524)
-Agilent 4150 TapeStation (4.1)

-Expression Console (1.4)
-GeneTrail (3.2)
-ClustVis (2.0)

For manuscripts utilizing custom algorithms or software that are central to the research but not yet described in published literature, software must be made available to editors and reviewers. We strongly encourage code deposition in a community repository (e.g. GitHub). See the Nature Portfolio guidelines for submitting code & software for further information.

Data

Policy information about availability of data

All manuscripts must include a data availability statement. This statement should provide the following information, where applicable:

- Accession codes, unique identifiers, or web links for publicly available datasets
- A description of any restrictions on data availability
- For clinical datasets or third party data, please ensure that the statement adheres to our policy

All data analysed in this study are included in this article and supplementary information, or have been made available in public repositories. Raw microarray data are available from the Gene Expression Omnibus (GEO) repository (GSE235670) (https://www.ncbi.nlm.nih.gov/geo/query/acc.cgi?acc=GSE235670). Raw sequence reads data are available from the Sequence Read Archive (SRA) repository (PRJNA992677) (https://www.ncbi.nlm.nih.gov/bioproject/PRJNA992677/). Flow cytometry data have been deposited in the Mendeley Data under DOI: 10.17632/b5bybfddnn.1 [https://data.mendeley.com/datasets/b5bybfddnn/1].

Research involving human participants, their data, or biological material

Policy information about studies with human participants or human data. See also policy information about sex, gender (identity/presentation), and sexual orientation and race, ethnicity and racism.

Reporting on sex and gender	n.a
Reporting on race, ethnicity, or other socially relevant groupings	n.a.
Population characteristics	n.a.
Recruitment	n.a.
Ethics oversight	n.a.

Note that full information on the approval of the study protocol must also be provided in the manuscript.

Field-specific reporting

Please select the one below that is the best fit for your research. If you are not sure, read the appropriate sections before making your selection.

☒ Life sciences ☐ Behavioural & social sciences ☐ Ecological, evolutionary & environmental sciences

For a reference copy of the document with all sections, see nature.com/documents/nr-reporting-summary-flat.pdf

Life sciences study design

All studies must disclose on these points even when the disclosure is negative.

Sample size	We did not use a priori statistical methods to determine the sample size. Sample size sufficiency was based on previous experiments from our laboratory and others: Gearty, S. V. et al. An autoimmune stem-like CD8 T cell population drives type 1 diabetes. Nature 602, 156–161 (2022).
Data exclusions	No data were excluded when analyzing data. All analyzed mice and samples were included for analysis.
Replication	All data were reproduced and compiled from independent experiments in figures and statistical analysis.
Randomization	Mice (wild type; heterozygous KO; homozygous KO) were housed in co-housed conditions after weaning to minimize the effect of different gut microbiota composition on diabetes development. The number of WT, hetero KO, homo KO mice was balanced in co-housing cages. Mice receiving donor splenocytes of different origins were randomly co-house in the same cage.
Blinding	We were not blinded to the allocation of mice, samples, and data analyses, to balance the co-housed conditions and to minimize the effect of different gut microbiota composition.

Reporting for specific materials, systems and methods

We require information from authors about some types of materials, experimental systems and methods used in many studies. Here, indicate whether each material, system or method listed is relevant to your study. If you are not sure if a list item applies to your research, read the appropriate section before selecting a response.

Materials & experimental systems

n/a	Involved in the study
☐	☒ Antibodies
☐	☒ Eukaryotic cell lines
☒	☐ Palaeontology and archaeology
☐	☒ Animals and other organisms
☒	☐ Clinical data
☒	☐ Dual use research of concern
☒	☐ Plants

Methods

n/a	Involved in the study
☒	☐ ChIP-seq
☐	☒ Flow cytometry
☒	☐ MRI-based neuroimaging

Antibodies

Antibodies used

Target name / Clone name / Manufacture / Catalogue No. / Lot No.

FITC anti-CD8a / 53-6.7 / BioLegend / 100706 / B329760
FITC anti-CD62L / MEL-14 / BioLegend / 104406 / B324091
FITC anti-CD4 / RM4-5 / eBioscience / 11-0042-81 / 2278300
FITC anti- RT1B(I-Ag7) / OX-6 / BioLegend / 205305 / B293077
PE anti-CD4 / GK1.5 / BioLegend / 100408 / B315274
PE anti-CD3 / 500A2 / eBioscience / 12-0033-81 / 1911672
PE anti-FOXP3 / MF-14 / BioLegend / 126403 / B350966
PE anti-IFN-γ / XMG1.2 / BioLegend / 505807 / B303388
PE anti-Ki67 / 16A8 / BioLegend / 652403 / B293052
PE anti-T-BET / 4B10 / BioLegend / 644809 / B347808
PE anti-BLIMP-1 / 5E7 / BioLegend / 150005 / B377137
PE anti-CD40 / 3/23 / BioLegend / 124609 / B353789
PE anti-CD11b / M1/70 / eBioscience / 12-0112-82 / E014719
PE anti-CD3 / 500A2 / eBioscience / 12-0033-81 / 1911672
PE anti-CD44 / 1M7 / eBioscience / 12-0441-81 / E028485
PE anti-TCF1 / C63D9/ Cell Signaling Technology / C63D9 / 9
PE AnnexinV/BioLegend/640907/B405461
PerCP Cy5.5 anti-CD3 / 17A2 / BioLegend / 100217 / B351063
PerCP Cy5.5 anti-GZMB / QA16A02 / BioLegend / 372211 / B357973
PerCP Cy5.5 anti-CD44 / 1M7 / BioLegend / 103032 / B213817
PerCP Cy5.5 anti-CD19 / 1D3 / eBioscience / 45-0193-80 / 4300340
APC anti-CD19 / 1D3 / BioLegend / 152409 / B285507
APC anti-CD44 / 1M7 / BioLegend / 103012 / B325365
APC anti-CD25 / PC61 / BioLegend / 102011 / B351503
APC anti-CXCR3 / CXCR3-173 / BioLegend / 126511 / B391488
APC anti-CD86 / GL-1 / BioLegend / 105011 / B346111
APC anti-CD103 / 2E7 / BioLegend / 121413 / B356817
APC anti-TCR γ/δ / GL3 / BioLegend / 118116 / B215599
APC anti-H-2Db / KH95 / BioLegend / 111513 / B351983
APC MHC Tetramer H-2Kd / - / MBL / TB-M552-2 / T2110004
Alexa Fluor 647 anti-IL17A / TC11-18H10 / BD Biosciences / 560184 / 3263593
APC-Cy7 anti-CD3 / 17A2 / BioLegend / 100222 / B324939
APC-Cy7 anti-CD45 / 30-F11 / BioLegend / 103116 / B345209
APC-Cy7 anti-CD8a / 53-6.7 / BioLegend / 100713 / B374507
PE-Cy7 anti-TCR γ/δ / GL3 / BioLegend / 118124 / B291445
PE-Cy7 anti-CD8a / 53-6.7 / BioLegend / 100721 / B364216
PE-Cy7 anti-CD4 / RM4-5 / BioLegend / 100527 / B281901
PE-Cy7 anti-CD11c / N418 / eBioscience / 25-0114-82 / 2295639
PE-Cy7 anti-EOMES / Dan11mag / eBioscience / 25-4875-80 / 2511279
BV421 anti-H2-Kd / SF-1.1 / BioLegend / 116623 / B294310
BV421 anti-CXCR3 / CXCR3-173 / BioLegend / 126521 / B315428
BV421 anti-CD103 / 2E7 / BioLegend / 121421 / B366374
BV450 anti-CD4 / RM4-5 / BD Biosciences / 560468 / 5351553
BV450 anti-CD11b / M1/70 / BD Biosciences / 560456 / 0050193
BV510 anti-CD45 / 30-F11 / BioLegend / 103134 / B314484
Purified anti-CD16/32 / 93 / BioLegend / 101302 / B320249

Dead cells were excluded using Zombie aqua fixable viability kit (BioLegend) or propidium iodide (Fujifilm).
AnnexinV staining was performed with AnnexinV binding buffer (BioLegend).

Antibodies information with dilution is shown in Supplementary Table 1.

Validation

We have relied on validation provided by suppliers for all primary antibodies we used in this study.

Validation

Target name / URL

FITC anti-CD8a / https://www.biolegend.com/ja-jp/products/fitc-anti-mouse-cd8a-antibody-153?GroupID=BLG2559
FITC anti-CD62L / https://www.biolegend.com/ja-jp/products/fitc-anti-mouse-cd62l-antibody-384?GroupID=BLG10714
FITC anti-CD4 / https://www.thermofisher.com/antibody/product/CD4-Antibody-clone-RM4-5-Monoclonal/11-0042-82
FITC anti- RT1B(I-Ag7) / https://www.biolegend.com/ja-jp/products/fitc-anti-rat-rt1b-antibody-5721?GroupID=GROUP27
PE anti-CD4 / https://www.biolegend.com/ja-jp/products/pe-anti-mouse-cd4-antibody-250?GroupID=BLG4745
PE anti-CD3 / https://www.thermofisher.com/antibody/product/CD3e-Antibody-clone-eBio500A2-500A2-Monoclonal/12-0033-82
PE anti-FOXP3 / https://www.biolegend.com/ja-jp/products/pe-anti-mouse-foxp3-antibody-4660?GroupID=BLG5706
PE anti-IFN-γ / https://www.biolegend.com/ja-jp/products/pe-anti-mouse-ifn-gamma-antibody-997?GroupID=GROUP24
PE anti-Ki67 / https://www.biolegend.com/ja-jp/products/pe-anti-mouse-ki-67-antibody-8134
PE anti-T-BET / https://www.biolegend.com/ja-jp/products/pe-anti-t-bet-antibody-6347?GroupID=BLG6433
PE anti-BLIMP-1 / https://www.biolegend.com/ja-jp/products/pe-anti-mouse-blimp-1-antibody-12328?GroupID=BLG14433
PE anti-CD40 / https://www.biolegend.com/ja-jp/clone-search/pe-anti-mouse-cd40-antibody-4983?GroupID=BLG5890
PE anti-CD11b / https://www.thermofisher.com/antibody/product/CD11b-Antibody-clone-M1-70-Monoclonal/12-0112-82
PE anti-CD3 / https://www.thermofisher.com/antibody/product/CD3e-Antibody-clone-eBio500A2-500A2-Monoclonal/12-0033-82
PE anti-CD44 / https://www.thermofisher.com/antibody/product/CD44-Antibody-clone-IM7-Monoclonal/12-0441-82
PE anti-TCF1 / https://www.cellsignal.jp/products/antibody-conjugates/tcf1-tcf7-c63d9-rabbit-mab-pe-conjugate/14456
PE AnnexinV / https://www.biolegend.com/ja-jp/productstab/pe-annexin-v-8145
PerCP Cy5.5 anti-CD3 / https://www.biolegend.com/ja-jp/products/percp-cyanine5-5-anti-mouse-cd3-antibody-5596?GroupID=BLG242
PerCP Cy5.5 anti-GZMB / https://www.biolegend.com/ja-jp/products/percp-cyanine5-5-anti-humanmouse-granzyme-b-recombinant-antibody-15597?GroupID=GROUP28
PerCP Cy5.5 anti-CD44 / https://www.biolegend.com/ja-jp/products/percp-cyanine5-5-anti-mouse-human-cd44-antibody-5605?GroupID=BLG10248
PerCP Cy5.5 anti-CD19 / https://www.thermofisher.com/antibody/product/CD19-Antibody-clone-eBio1D3-1D3-Monoclonal/45-0193-82
APC anti-CD19 / https://www.biolegend.com/ja-jp/products/apc-anti-mouse-cd19-antibody-13680
APC anti-CD44 / https://www.biolegend.com/ja-jp/products/apc-anti-mouse-human-cd44-antibody-312?GroupID=BLG10425
APC anti-CD25 / https://www.biolegend.com/ja-jp/products/apc-anti-mouse-cd25-antibody-420?GroupID=BLG10428
APC anti-CXCR3 / https://www.biolegend.com/ja-jp/products/apc-anti-mouse-cd183-cxcr3-antibody-4683?GroupID=BLG5657
APC anti-CD86 / https://www.biolegend.com/ja-jp/products/apc-anti-mouse-cd86-antibody-2896?GroupID=BLG10719
APC anti-CD103 / https://www.biolegend.com/ja-jp/products/apc-anti-mouse-cd103-antibody-4914?GroupID=BLG4646
APC anti-TCR γ/δ / https://www.biolegend.com/ja-jp/search-results/apc-anti-mouse-tcr-gamma-delta-antibody-6061?GroupID=BLG3687
APC anti-H-2Db / https://www.biolegend.com/ja-jp/products/apc-anti-mouse-h-2d-b-antibody-11947?GroupID=GROUP20
APC MHC Tetramer H-2Kd / https://ruo.mbl.co.jp/bio/dtl/T/index.html?pcd=TB-M552-2
Alexa Fluor 647 anti-IL17A / https://www.bdbiosciences.com/ja-jp/products/reagents/flow-cytometry-reagents/research-reagents/single-color-antibodies-ruo/alexa-fluor-647-rat-anti-mouse-il-17a.560184
APC-Cy7 anti-CD3 / https://www.biolegend.com/ja-jp/explore-new-products/apc-cyanine7-anti-mouse-cd3-antibody-6068?GroupID=BLG242
APC-Cy7 anti-CD45 / https://www.biolegend.com/ja-jp/products/apc-cyanine7-anti-mouse-cd45-antibody-2530?GroupID=BLG1932
APC-Cy7 anti-CD8a / https://www.biolegend.com/ja-jp/products/apc-cyanine7-anti-mouse-cd8a-antibody-2269?GroupID=BLG279
PE-Cy7 anti-TCR γ/δ / https://www.biolegend.com/ja-jp/products/pe-cyanine7-anti-mouse-tcr-gamma-delta-antibody-7822?GroupID=BLG8973
PE-Cy7 anti-CD8a / https://www.biolegend.com/ja-jp/productstab/pe-cyanine7-anti-mouse-cd8a-antibody-1906?GroupID=BLG2559
PE-Cy7 anti-CD4 / https://www.biolegend.com/ja-jp/products/pe-cyanine7-anti-mouse-cd4-antibody-1932?GroupID=BLG4211
PE-Cy7 anti-CD11c / https://www.thermofisher.com/antibody/product/CD11c-Antibody-clone-N418-Monoclonal/25-0114-82
PE-Cy7 anti-EOMES / https://www.thermofisher.com/antibody/product/EOMES-Antibody-clone-Dan11mag-Monoclonal/25-4875-82
BV421 anti-H2-Kd / https://www.biolegend.com/ja-jp/products/brilliant-violet-421-anti-mouse-h-2kd-antibody-11960?Clone=SF1-1.1
BV421 anti-CXCR3 / https://www.biolegend.com/ja-jp/products/brilliant-violet-421-anti-mouse-cd183-cxcr3-antibody-7159?GroupID=BLG10642
BV421 anti-CD103 / https://www.biolegend.com/ja-jp/products/brilliant-violet-421-anti-mouse-cd103-antibody-7329?GroupID=BLG4648
BV450 anti-CD4 / https://www.bdbiosciences.com/ja-jp/products/reagents/flow-cytometry-reagents/research-reagents/single-color-antibodies-ruo/v450-rat-anti-mouse-cd4.560468
BV450 anti-CD11b / https://www.bdbiosciences.com/ja-jp/products/reagents/flow-cytometry-reagents/research-reagents/single-color-antibodies-ruo/v450-rat-anti-cd11b.560455
BV510 anti-CD45 / https://www.biolegend.com/ja-jp/products/brilliant-violet-510-anti-mouse-cd45-antibody-7995
Purified anti-CD16/32 / https://www.biolegend.com/ja-jp/products/purified-anti-mouse-cd16-32-antibody-190?GroupID=BLG9237

Eukaryotic cell lines

Policy information about cell lines and Sex and Gender in Research

Cell line source(s)

NIT-1: We obtained NIT-1 cells from ATCC (CRL-2055). NIT-1 is a pancreatic beta cell line that was isolated from the islet of langerhans of a 10-week-old, female mouse with insulinoma.

Authentication

The cell lines was not authenticated.

Mycoplasma contamination

Contamination of Mycoplasma was not detected.

Commonly misidentified lines
(See ICLAC register)

The cell lines was not listed in Commonly misidentified lines.

Animals and other research organisms

Policy information about studies involving animals; ARRIVE guidelines recommended for reporting animal research, and Sex and Gender in Research

Laboratory animals	NOD/shiJcl mice were purchased from CLEA Japan. NOD/SCID mice were purchased from the Jackson laboratory Japan. NY8.3-NOD mice were provided by S. Akazawa and N. Abiru (Nagasaki University, Nagasaki, Japan) with permission from the Jackson laboratory. Tyk2 knockout mice were provided by K. Shimoda (University of Miyazaki, Miyazaki, Japan). Tyk2KO/NOD mice and Tyk2KO/NY8.3-NOD mice were generated in-house. All mice were maintained on a 12-hour light/dark cycle in a temperature-controlled facility under specific pathogen-free conditions at 23±2C with 50±10% humidity, and provided with sterile food and water ad libitum. SCID mice were fed with sterile water. We used 6-7w, 10-12w, 14w, 24w of age mice as indicated in the figure legends.
Wild animals	None.
Reporting on sex	We used female mice in all experiments because the incidence of autoimmune diabetes is more prevalent in female NOD mice.
Field-collected samples	None.
Ethics oversight	All animal experiments were approved by the Saga University Animal Care and Use Committee and conducted in accordance with the regulations on animal experimentation at Saga University (Approval No. A2019-047-0, A2021-007-0, A2021-023-0, A2022-044-0, A2022-045-1).

Note that full information on the approval of the study protocol must also be provided in the manuscript.

Flow Cytometry

Plots

Confirm that:

☒ The axis labels state the marker and fluorochrome used (e.g. CD4-FITC).

☒ The axis scales are clearly visible. Include numbers along axes only for bottom left plot of group (a 'group' is an analysis of identical markers).

☒ All plots are contour plots with outliers or pseudocolor plots.

☒ A numerical value for number of cells or percentage (with statistics) is provided.

Methodology

Sample preparation	To isolate immune cells from the LNs, thymus, or spleen, tissues were minced and enzymatically dissociated with 1 mg/mL collagenase D and 25 U/mL DNase I in DMEM for 30 min at 37°C. The digested tissues were filtered through a 70-µm cell strainer with a syringe plunger. Red blood cell lysis was performed by incubation with ammonium-chloride-potassium lysis buffer for 4 min on ice. Isolated cells were preincubated with CD16/32 blocker for 5 minutes at 4°C to prevent nonspecific staining, and stained for 20 min at 4°C with antibodies. We added 1 mg/mL propidium iodide to the cell suspension to exclude dead cells.<br>To isolate immune cells from the pancreas, pancreases were perfused with 1 mg/mL collagenase D and 0.5 mg/mL Trypsin inhibitor and 25 U/mL DNase I through the bile duct. The perfused pancreas was removed carefully from the intestine, stomach, spleen, and LNs, and dissociated for 30 min at 37C. Then, 5 mL of DMEM containing 10% FBS and 1% PcSM was added to the dissociated pancreas and shaken for 10 seconds to completely dissociate the tissue. The digested pancreas was filtered through a 70-µm cell strainer with a syringe plunger. Red blood cell lysis was performed by incubation with ammonium-chloride-potassium lysis buffer for 4 min on ice. The digested pancreas was centrifuged and resuspended in 40% Percoll layered on 70% Percoll, and centrifuged at 2200 rpm for 20 min. Cells were collected from the Percoll interface. Isolated cells were preincubated with CD16/32 blocker for 5 minutes at 4°C to prevent nonspecific staining, and stained for 20 min at 4C with antibodies. We added 1 mg/mL propidium iodide to the cell suspension to exclude dead cells.<br>To stain IFN-γ and IL-17A, cells were stimulated with 25 ng/mL PMA and 1 mg/mL ionomycin for 6 hours at 37C. Then, 10 mg/mL Brefeldin A was added for the last 5 hours of incubation. Intracellular staining was performed using a Cytofix/Cytoperm Fixation/Permeabilization Solution kit according to the manufacturer's instructions. The intracellular staining of FOXP3, KI67, T-BET, EOMES, TCF-1, GZMB, and BLIMP-1, was performed using the Foxp3/Transcription factor staining buffer set according to the manufacturer's instructions. Dead cells were excluded using a Zombie Aqua fixable viability kit (BioLegend).
Instrument	Data were obtained using FACSVerse (BD Bioscience) and MA900 (Sony).
Software	Data were analyzed using FlowJo (10.6.1).
Cell population abundance	The abundance of cell populations were calculated by FlowJo software. The absolute number of cells was calculated by multiplying the number of original cell suspensions by the parentage of the cell population calculated by FlowJo.

Gating strategy

First, live cells were defined by propidium iodide or Zombie aqua dye staining. Single cells were selected by SSC-W and SSC-H plots. Single cells were selected again by FSC-W and FSC-H plots. Gating strategies were provided in the supplementary figure 2.

☒ Tick this box to confirm that a figure exemplifying the gating strategy is provided in the Supplementary Information.
